# CircUBXN7 promotes macrophage infiltration and renal fibrosis associated with the IGF2BP2-dependent SP1 mRNA stability in diabetic kidney disease

**DOI:** 10.3389/fimmu.2023.1226962

**Published:** 2023-09-06

**Authors:** Ziyue Lin, Dan Lv, Xiaohui Liao, Rui Peng, Handeng Liu, Tianhui Wu, Keqian Wu, Yan Sun, Zheng Zhang

**Affiliations:** ^1^ Department of Nephrology, The Second Affiliated Hospital of Chongqing Medical University, Chongqing, China; ^2^ Department of Cell Biology and Genetics, Chongqing Medical University, Chongqing, China; ^3^ Department of Bioinformatics, Chongqing Medical University, Chongqing, China; ^4^ Center of Teaching and Learning, Chongqing Medical University, Chongqing, China

**Keywords:** diabetic kidney disease, CircUBXN7, IGF2BP2, macrophage infiltration, epithelial-mesenchymal transition, tubulointerstitial fibrosis

## Abstract

**Introduction:**

Inflammatory cell infiltration is a novel hallmark of diabetic kidney disease (DKD), in part, by activated macrophages. Macrophage-to-tubular epithelial cell communication may play an important role in renal fibrosis. Circular RNAs (circRNAs) have been reported in the pathogenesis of various human diseases involving macrophages activation, including DKD. However, the exact mechanism of circRNAs in macrophage infiltration and renal fibrosis of DKD remains obscure.

**Methods:**

In our study, a novel circRNA circUBXN7 was identified in DKD patients using microarray. The function of circUBXN7 *in vitro* and *in vivo* was investigated by qRT-PCR, western blot, and immunofluorescence. Finally, a dual-luciferase reporter assay, ChIP, RNA pull-down, RNA immunoprecipitation and rescue experiments were performed to investigate the mechanism of circUBXN7.

**Results:**

We demonstrated that the expression of circUBXN7 was significantly upregulated in the plasma of DKD patients and correlated with renal function, which might serve as an independent biomarker for DKD patients. According to investigations, ectopic expression of circUBXN7 promoted macrophage activation, EMT and fibrosis *in vitro*, and increased macrophage infiltration, EMT, fibrosis and proteinuria *in vivo*. Mechanistically, circUBXN7 was transcriptionally upregulated by transcription factor SP1 and could reciprocally promote SP1 mRNA stability and activation via directly binding to the m6A-reader IGF2BP2 in DKD.

**Conclusion:**

CircUBXN7 is highly expressed in DKD patients may provide the potential biomarker and therapeutic target for DKD.

## Introduction

Diabetes affects more than 450 million individuals globally, of which 95% have type 2 diabetes. Diabetic kidney disease (DKD) is a progressive renal disease secondary to diabetes, and has been proven to be the leading cause of end-stage renal disease (1, 2). DKD progression involves renal inflammation, glomerular hypertrophy, proteinuria, glomerulosclerosis, interstitial fibrosis, interstitial infiltrating immune cells and renal failure (3–7). Tubulointerstitial fibrosis is a prevalent final pathway leading to progressive renal damage in DKD (8). The emergence of epithelial-mesenchymal transition (EMT) in the renal tubule epithelial cells is linked with tubulointerstitial fibrosis during renal injury (9). The molecular mechanisms underlying renal EMT and tubulointerstitial fibrosis in DKD are still largely unclear, despite extensive research, thus hindering the development of diagnosis and treatment of DKD.

Mounting evidence supports the involvement of immune cells as a critical contributor in DKD pathogenesis, particularly through macrophages (10, 11). Macrophages infiltration to the kidney promoted macrophage activation and tubular epithelial cells injury via tubular epithelial cell-to-macrophage communication to exacerbate DKD (12). Upregulated VCAM1 expression in renal tubular cells promoted interstitial fibrosis via interacting with infiltrated immune cells in DKD (7). Moreover, Piezo1 deletion suppressed macrophage inflammation and consequently restrained macrophage-induced renal fibrosis and epithelial-mesenchymal transition (13). Thus, macrophages play important roles in renal fibrosis. However, the specific mechanism in DKD is not clear.

Circular RNAs (circRNAs) are a form of single-stranded RNA transcripts lacking a 5′cap or 3′poly A tail but possessing a covalently bonded circular structure created from pre-mRNAs via back-splicing (14). The distribution and role of circRNAs have been widely reported in various diseases (15–17), suggesting circRNAs may be novel clinical diagnostic markers or therapeutic targets (18). Prior research has shown that dysregulated circRNAs expression is related to DKD, regulating renal inflammation, glomerular hypertrophy, tubulointerstitial fibrosis, and sclerosis (19). For example, it has been reported that circRNA_010383 (20) and circ_DLGAP4 (21) have significant roles in renal fibrosis development in DKD. CircACTR2 activates macrophage inflammation to stimulate macrophage-induced EMT and fibrosis of renal tubular epithelial cells (22). However, little is known about the possible significance of circRNAs in renal fibrosis of DKD, and the potential mechanism of circRNAs remains mainly elusive in DKD.

In our study, a series of differentially expressed circRNAs were identified in the plasma of DKD patients via circRNAs microarray. Here, we found circUBXN7 was significantly upregulated in HK-2 cells cultivated with high glucose and plasma of DKD patients as well as correlated with renal function, which might act as an independent biomarker for DKD patients. Next, we identified that circUBXN7 showed transcriptional upregulation using SP1 under high glucose conditions and regulated macrophage activation and fibrosis of HK-2 cells to facilitate DKD progression *in vivo* and *in vitro*. Other mechanism research revealed that circUBXN7 formed an RNA-protein complex with IGF2BP2 and SP1, activating mRNA stability and expression of SP1, and aggravating the program of EMT and renal tubulointerstitial fibrosis in DKD. Our results provide a novel molecular mechanism by which high glucose-induced circUBXN7 contributes to DKD progression, suggesting that circUBXN7 could be used as a possible biomarker and therapeutic target for DKD.

## Materials and methods

### Plasma sample

The plasma of DKD patients (n=49) and healthy people (n=54) were registered in our research, which was collected from the Second Affiliated Hospital of Chongqing Medical University (Chongqing, China). Inclusion criteria: 1. Type 2 diabetes-diagnosed patients, 2. patients with urine albumin/creatinine ratio >40 mg/g, 3. Patient without other diseases such as ketoacidosis, malignancy, trauma, infection, hepatitis, or autoimmune disease. The study was conducted with agreement from the Ethical Committee of the Second Affiliated Hospital of Chongqing Medical College (2018146). All participants gave their written informed consent.

### CircRNA microarray analysis

Plasma circRNAs from DKD patients and healthy controls were obtained for microarray analysis (https://www.ncbi.nlm.nih.gov/geo/query/acc.cgi?acc=GSE231923). The RNA of plasma sample preparation was done regarding Arraystar protocols. Finally, Arraystar human circRNA Array V2.0 was utilized to hybridize labeled RNAs, and the circRNAs were analyzed using circRNA microarrays (Aksomics, China). We defined the differentially expressed circRNAs with the criteria of p values < 0.05.

### Bioinformation analysis

The origin of circUBXN7 was acquired from UCSC Genome Browser (https://genome.ucsc.edu/index.html). UBXN7 promoter sequences were acquired from National Center for Biotechnology Information (NCBI) sequence database. Transcription factors and their potential and specific binding sites within UBXN7 promoter were predicted by PROMO (http://alggen.lsi.upc.es/cgi-bin/promo_v3/promo/promoinit cgi?dirDB=TF_8.3) and JASPAR (http://jaspar.genereg.net/), respectively.

Enrichment analysis was conducted utilizing DAVID online database (https://david.ncifcrf.gov/summary.jsp) by setting a P-value < 0.05 as a filtering criterion. The GO enrichment analysis includes three components molecular function (MF), biological process (BP), and cellular component (CC).

Alphafold software was utilized to predict IGF2BP2 protein structure, and the molecular modeling Rosetta was utilized to predict and model the binding of circUBXN7 and IGF2BP2.

m6A2Target (http://m6A2target.canceromics.org/) is an integrated database including target genes corresponding to three classes of m6A (writers, erasers, and readers). We explored IGF2BP2 m^6^A downstream targets through the m6A2Target database.

### Cell culture

Human renal tubular epithelial cells (HK-2) were preserved in our laboratory. HK-2 was routinely cultivated in DMEM/F12 media (Gibco, CA, USA) with 10% fetal bovine serum in a humidified 5% CO2 incubator at 37°C. Many previous studies have demonstrated that high glucose (HG) conditions (25 mmol/L glucose) induce DKD state, while low glucose (LG) conditions (5.5 mmol/L glucose and 19.5 mmol/L mannitol) induce a normal growth status.

### Co-culture of macrophages and HK-2 cells

The macrophages used in the study were derived from the mouse peritoneal macrophages (RAW264.7), which were cultivated in DMEM medium (Gibco, CA, USA) with 10% fetal bovine serum in a humidified 5% CO_2_ incubator at 37 °C. The transwells were used to realize co-culture of macrophages and HK-2 cells. The 0.4 μm aperture PET semipermeable membrane blocked the upper and lower chambers, allowing only small molecules to pass through, but not cells. HK-2 cells (1×10^5^) from the OE-NC, OE-circ, si-NC, and si-circ groups were seeded in the upper chamber of the transwell plate, while macrophages (4×10^5^) were seeded in the 6-well plate of the lower chamber. Then upper ventricular cells were stimulated with LG/HG medium for 24 and 48 hours, and lower ventricular macrophages were collected for further experiments. Cell lines were tested for mycoplasma contamination using GMyc-PCR Mycoplasma Test Kit (Yeasen biotech, 40601ES20).

### RNA extraction and qRT-PCR assays

Total RNA was extracted from HK-2 cells grown under LG or HG utilizing Trizol reagent (Takara, Dalian, China) and transcribed into cDNA using PrimeScript RT Reagents (Takara) as per manufacturer guidelines. On a Bio-Rad CFX96 system, the TB Green Premix Ex Taq (Takara) was utilized to conduct quantitative real-time PCR (qRT-PCR) (Bio-Rad, CA, USA). The relative gene expression was determined utilizing 2–ΔΔCT and standardized by β-actin. **Table S1** included all primer sequences.

### Lentivirus packaging, siRNAs and cell transfection

Amplification and cloning of the full circUBXN7 liner sequence into the lentivirus vector (LV) pLC5-ciR (Genechem, Shanghai, China) enabled the formation of a circUBXN7 overexpression vector or OE-circ. HK-2 cells were transfected using a lentivirus vector. After 48 h, cells infected with lentivirus were collected and screened with puromycin. RiboBio (Guangzhou, China) generated three siRNAs targeting the back-splicing locus of circUBXN7 and negative control (si-NC), termed si-circ and si-NC. Based on si-circ2, we designed and integrated circUBXN7-targeting shRNA into U6-MCS-PGK-EGFP lentiviral vectors (Hanbio, Shanghai, China), termed sh-circ. SP1 and IGF2BP2 were cloned into pcDNA3.1 after their full lengths were amplified from cDNA (RiboBio, Guangzhou, China). RiboBio developed and produced the siRNAs and si-NC targeting SP1 and IGF2BP2 (Guangzhou, China). As per manufacturer guidelines, Lipofectamine 2000 (Invitrogen, Carlsbad, CA, USA) was utilized for cells transfection. **Table S2** listed siRNA sequences.

### Nuclear-cytoplasmic fractionation

Nuclear and cytoplasmic RNA of HK-2 cells were isolated utilizing PARIS™ Kit (Invitrogen). 2–ΔΔCT was utilized to standardize GAPDH data (cytoplasmic control) and U6 (the nuclear control).

### RNase R and actinomycin D assay

HK-2 cell total RNA (2 μg) was incubated at 37°C for 10, 20, 30, or 40 min with or without RNase R (Thermo Fisher Scientific, MA, USA). The β-actin, circUBXN7, and linearUBXN7 expressions were analyzed using qRT-PCR. Actinomycin D (5 μg/mL) or DMSO (Sigma Aldrich, St. Louis, MO, USA) was applied to HK-2 cells to assess the stability of β-actin, circUBXN7, and linearUBXN7. qRT-PCR was utilized to determine RNA stability.

### Western blot

HK-2 cells were added in RIPA lysis buffer containing PMSF, and BCA Protein Assay Kit (KeyGEN, BioTECH, China) was utilized for protein quantification. Proteins were then resolved on an SDS-PAGE gel (8% or 10%) and translocated to PVDF membranes. The membranes were treated with primary antibodies at 4°C for 24 h after being blocked with 5% skim milk at room temperature (RT) for 2 h. The primary antibodies included β-actin (1:5000), E-cadherin (1:5000), Vimentin (1:5000), α-SMA (1:3000), Collagen I (1:5000), TGFβ1 (1:1000), iNOS (1:2000), CD86 (1:2000), IL-6 (1:1000), TNFα (1:1000), SP1 (1:1000) (Abcam), IGF2BP2 (1:1000, Proteintech). Subsequently, membranes were incubated with a secondary antibody solution containing either goat (anti-rabbit or anti-mouse) HRP-IgG for 1.5 h. Afterward, protein concentration was measured using an enhanced chemiluminescence (ECL) kit (Pierce Biotechnology, USA). ImageJ was utilized to evaluate the semiquantitative band data.

### Fluorescence *in situ* hybridization

GenePharma synthesized biotin-labeled probes for the circUBXN7 junction site (5’-GAACTTCTTCTTGTTTCAAAGCTGCCTTT-3’). FISH analysis was conducted utilizing a FISH kit as per manufacturer guidelines, followed by counterstaining with DAPI.

### Immunofluorescence analysis

After 48 h in culture, cells were fixed with paraformaldehyde (PFA)for 30 min, treated with 0.5% Triton X-100 for 10 min, blocked with 3% goat serum for 1 h, then incubated overnight with antibodies, including E-cadherin (1:250), Vimentin (1:250), α-SMA (1:150), Collagen I (1:250), TGFβ1 (1:50), F4/80 (1:250), SP1 (1:150, Abcam) on sterile cover glass slides in a 24-well plate. After 1 h at dark condition, cells were incubated with a secondary immunofluorescence antibody. Next, DAPI was supplemented to the cells for 10 min.

### Dual-luciferase reporter assay

UBXN7 promoter containing three presumptive binding sites of SP1. The 100bp region before and after the P1, P2, or P3 sites of UBXN7 promoter and its respective mutations were synthesized and inserted into a pGL3-basic luciferase reporter vector (Ribo Biotech, Guangzhou, China) based on experimental design for SP1 binding sites. Regarding manufacturer protocol, the above vectors were co-transfected with pcDNA3.1-SP1 vector and renilla luciferase plasmids, respectively. After two days of transfection, the luciferase activity was evaluated utilizing Dual-Luciferase Reporter Assay (Hanbio, Shanghai, China) as per manufacturer guidelines and normalized to the activity of renilla luciferase.

### Chromatin immunoprecipitation

SP1-specific antibodies (Abcam), an IgG control (CST), and the SimpleChIP^®^ Enzymatic Chromatin IP Kit (CST) were employed to perform chromatin immunoprecipitation. The primer sequences were all provided in **Table S1**.

### RNA pull-down assay

The circUBXN7 junction site was the target of biotin-labeled probes (5’- GAACTTCTTCTTGTTTCAAAGCTGCCTTT-3’-Biotin). A Magnetic RNA-Protein Pull-Down Kit (Thermo Fisher) was utilized to do the RNA pull-down at the specified conditions.

### RNA immunoprecipitation assay

RNA Immunoprecipitation Kit (Geneseed) was utilized to detect interactions among IGF2BP2 and circUBXN7, IGF2BP2, and SP1 with antibodies specific for IGF2BP2 (Abcam), IgG control (CST), and all experimental steps were performed according to the RIP procedure. Western blot was used to detect IGF2BP2 proteins level, and simultaneously qRT-PCR was employed to analyze the enrichment status of circUBXN7 and SP1.

### Transcription factor activation array

Transcription factor activity assays are performed according to TF Activation Profiling Plate Array I kit instructions (Signosis). Nuclear extract/probe mixture (15 µg nuclear extract and 3 µl biotin-labeled probe mix I) incubated at RT for 30 min. Free probes were isolated from TF/probe complexes using a spin column purification procedure, although individual probes can locate their target TF and form complexes with it. The hybridization plate was used to separate the bound probes from the complex for analysis. The captured DNA probes were further identified with Streptavidin-HRP Conjugate and then assessed with a chemiluminescent plate reader (Veritas microplate luminometer). On a microplate luminometer, luminescence is measured in relative light units (RLUs).

### Animal model

The male mice of db/db were obtained from the Chinese Nanjing Biomedical Research Institute with C57BL/BKS background (Leprdb/+Leprdb). As we previously reported (23), blood glucose, albuminuria, and creatinine levels were observed in db/db and db/m mice, and 8-week-old db/db mice were regarded to be in the early stages of DKD. These DKD mice were divided into DKD + LV-NC group (NC), DKD + LV-sh-circUBXN7 (sh-circ), and DKD + LV-OE-circUBXN7 group (OE-circ). n = 10 in each group. LV-NC, LV-sh-circUBXN7, and LV-OE-circUBXN7 (1x10^7^ TU/mL, 100 µL) were injected into DKD mice via the tail vein. Following four weeks of anesthesia with 50 mg/kg intraperitoneal sodium pentobarbital, all mice were sacrificed. The tissue specimens were fixed using 4% PFA or frozen in liquid nitrogen until further use. All animal studies and management meet Chongqing Medical University protocols. Chongqing Medical University Ethics Committee authorized the project (2022135).

### Blood and urine biochemistry assays

Using the HemoCue B-Glucose kit, the fasting blood glucose level was determined. Using the Creatinine Assay kit (sarcosine oxidase) (Nanjing, China), creatinine levels in the urine were measured. The mouse urine albumin ELISA kit was utilized to quantify urinary albumin. The urine albumin/creatinine ratio (UACR) was determined by calculating the urinary albumin/creatinine ratio.

### Histological staining

To evaluate morphological and pathological alterations, kidney tissue specimens treated with 4% PFA were fixed in paraffin and subsequently sliced to a thickness of 3 m. Under a light microscope, tissue slices were stained with hematoxylin and eosin (H&E), Masson, and periodic acid-silver (PAS) (24), respectively.

### Statistical analysis

GraphPad Prism 8.0 (GraphPad Software, San Diego, CA) was utilized in all statistical analyses. The presentation of all data is as mean± standard deviation (SD). As indicated, Student’s t-test or one-way ANOVA was employed for statistical comparisons. Pearson’s correlation test was utilized to assess correlations. P < 0.05 was regarded as statistically significant.

## Results

### Elevated circUBXN7 is correlated with renal function in DKD patients

**Figure 1:**
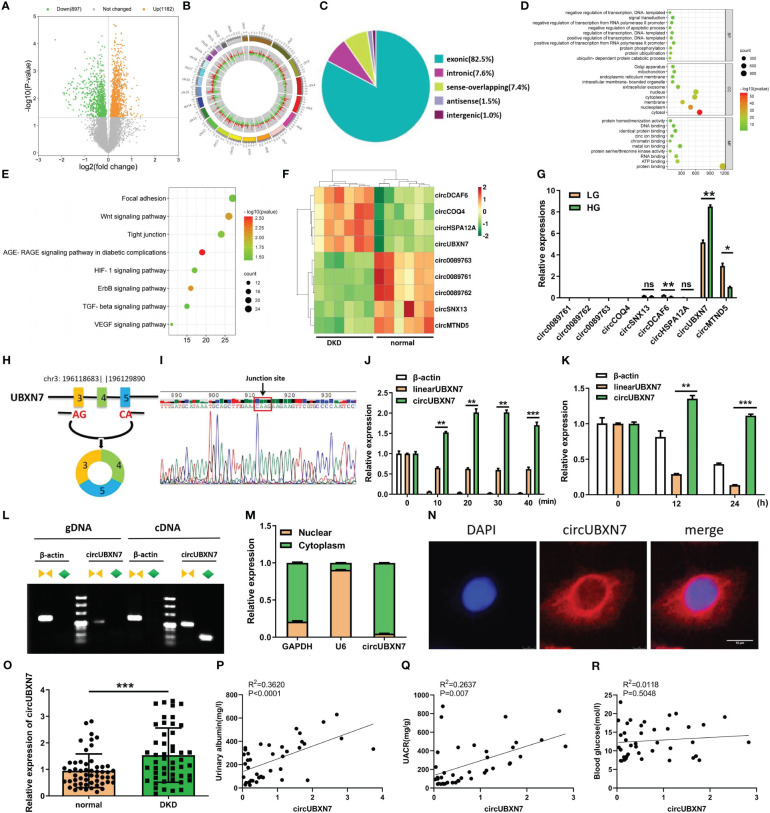
CircUBXN7 is validated and characterized in DKD patients and HK2 cells under high glucose. **(A)** 2079 circRNAs were found in the plasma of DKD patients (n=6) and healthy people (n=6) by Arraystar Human Circular RNA Microarray (P<0.05); **(B)** Chromosomal distribution of 2079 circRNAs in plasma of DKD patients and normal people; **(C)** The source of 2079 circRNAs was shown; **(D)** GO functional enrichment analysis of 2079 DKD-related circRNAs. BP, biological process; CC, cell components; MF, molecular function; **(E)** Eight EMT-related KEGG pathways enrichment results of 2079 circRNAs. The bubble size indicates the circRNAs number, and the color represents the p-value; **(F)** Nine differentially expressed circRNAs were associated with DKD, including four up-regulated and five down-regulated circRNAs (|FC|>1.5, FDR<0.05); **(G)** qRT-PCR analysis of circ0089761, circ0089762, circ0089763, circCOQ4, circSNX13, circDCAF6, circHSPA12A, circUBXN7 and circMTND5 relative expression among HKs cells cultured with high and low glucose; **(H)** Schematic diagram illustrated that circUBXN7 was generated by the circularization of exons 3, 4, and 5 of gene UBXN7 on chromosome 3; **(I)** Sanger sequencing of the back-splicing junction of circUBXN7; **(J)** The levels of circUBXN7 and linearUBXN7 mRNA of HK2 cells were detected following RNase R digestion at different time gradients by qRT-PCR; **(K)** The expressions of circUBXN7 and linearUBXN7 of HK2 cells were analyzed by qRT-PCR after treatment with actinomycin D (5 μg/mL); **(L)** RT-PCR and agarose gel electrophoresis showed that circUBXN7 was only detected by divergent primers from cDNA but not gDNA; **(M)** Nuclear-cytoplasmic fractionation assay displayed that circUBXN7 was mostly distributed in the cytoplasm of HK2 cells by qRT-PCR; **(N)** The localization of circUBXN7 was investigated in HK2 cells by FISH. Scale bar, 10 μm; **(O)** Relative expression of circUBXN7 detected by qRT-PCR in plasma of DKD patients (n=49) and normal people (n=54); **(P, Q)** Pearson correlation analysis indicated that circUBXN7 expression was strongly correlated with the Urinary albumin level **(P)**, R^2^ = 0.3620, P< 0.0001) and UACR (Q, R^2^ = 0.2637, P = 0.007); **(R)** There was no significant correlation between circUBXN7 expression and blood glucose level by Pearson correlation analysis (R^2^ = 0.0118, P= 0.5048). Data are shown as mean ± SD and representative of three independent experiments. *P < 0.05, **P < 0.01, ***P < 0.001, ns, no significance.

To determine if the special circRNA is present in DKD, we first detected circRNAs expression in the plasma of DKD patients (n=6) and healthy controls (n=6) by circRNAs microarray. According to the microarray data, we found 2079 DKD-related circRNAs, including 1182 up-regulated and 897 down-regulated (P<0.05) (**Figure 1A**). Through performing the analysis of chromosomal location of circRNAs, we found that the most circRNAs were located on chromosomes 1, 2, and 3 (**Figure 1B**). Next, we analyzed the sources of circRNAs and found exonic circRNAs accounted for 82.5%, intronic circRNAs accounted for 7.6%, sense overlapping circRNAs accounted for 7.4%, antisense circRNAs accounted for 1.5%, and intergenic circRNAs accounted for 1% (**Figure 1C**). The enrichment analysis of GO and KEGG presented the DKD-related circRNA target genes involved in protein binding, RNA binding, positive transcriptional control from RNA polymerase II promoter and signal transduction and DKD-related circRNA induced the transcriptional reprogramming of networks related to EMT-related signaling pathways (**Figures 1D, E**). Among the differentially expressed circRNA, nine representative circRNAs (|FC| > 1.5, FDR < 0.05) were chosen (**Figure 1F**) and further validated by qRT-PCR (**Figure 1G**). qRT-PCR results revealed that circUBXN7 was significantly elevated and the most abundant in HK-2 cells cultivated with high glucose. These results imply that circUBXN7 should be a DKD-related circRNA, and was stimulated by high glucose in HK-2 cells.

To explore the origin of circUBXN7, we found circUBXN7 was a circular transcript produced from back-splicing of exons 3, 4, and 5 of UBXN7 gene, which was positioned on human chromosome 3 with a 247nt-length using UCSC Genome Browser (**Figure 1H**). The cyclization site -CAAG- of circUBXN7 sequence was confirmed by sanger sequencing (**Figure 1I**). Next, circUBXN7 resisted the degradation of RNase R, while linearUBXN7 and β-actin mRNA were non-resistant to the degradation (**Figure 1J**). To confirm circUBXN7 stability, HK-2 cells were treated with actinomycin D, a RNA synthesis inhibitor, and discovered that circUBXN7 mRNA stability was significantly greater than linearUBXN7 mRNA stability (**Figure 1K**). The results of RT-PCR revealed that only circUBXN7, rather than linearUBXN7 and β-actin, could show amplification with divergent primers in cDNA (**Figure 1L**). Cytoplasmic/nuclear fractionation assay (**Figure 1M**) and FISH results (**Figure 1N**) revealed that circUBXN7 was dominantly located in the cytoplasm of HK-2 cells.

Additionally, circUBXN7 is significantly elevated in the plasma of DKD patients (**Figure 1O**; **Table 1**). Pearson correlation analysis demonstrated a positive relationship between circUBXN7 expression and urinary albumin (**Figure 1P**) or the ratio of urinary albumin to creatinine (UACR) (**Figure 1Q**) in plasma of DKD patients, while no significant relevance was found in circUBXN7 expression with blood glucose level (**Figure 1R**). Together, these findings suggest that circUBXN7 is a novel upregulated circRNA in DKD, and might be a diagnostic plasma marker for DKD.

**Table 1:** Clinical information of plasma samples.

	Normal control (n=54)	DKD patients (n=49)
Age(y)	46.50 ± 7.50	60.94 ± 10.57***
Sex(♂/♀)	20/34	29/20
Blood glucose(mmol/L)	4.75 ± 0.88	13.92 ± 5.86***
hemoglobin A1c(%)	5.07 ± 0.65	9.10 ± 2.34***
Urinary albumin(mg/L)	–	247.01 ± 247.15
Urine creatinine(umol/L)	–	8299.29 ± 4727.83
UACR(mg/g)	<30.00	315.93 ± 348.71

***P<0.0001.

### CircUBXN7 promotes macrophage activation and fibrosis of HK2 cells.

**Figure 2:**
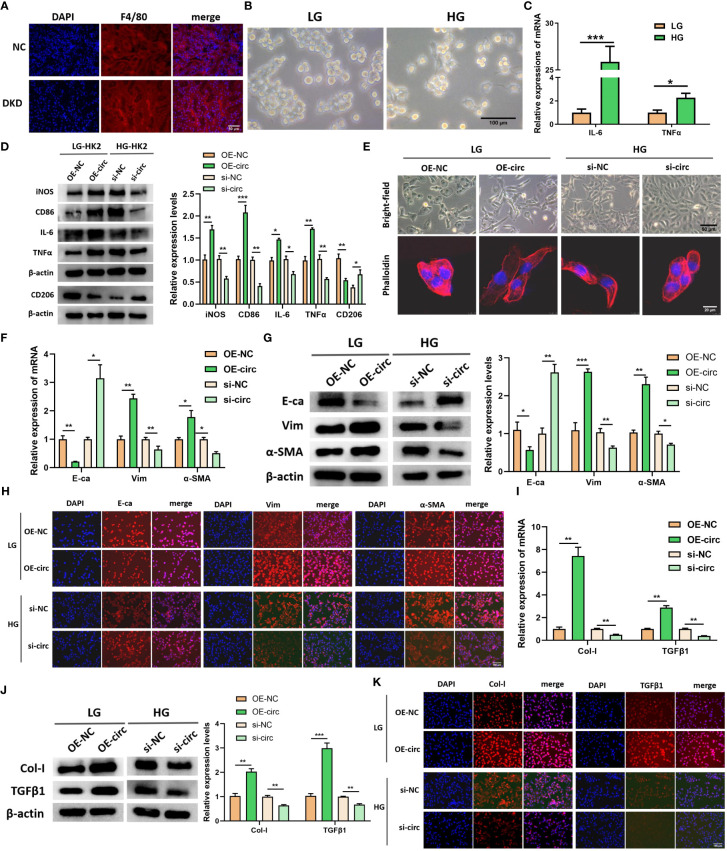
CircUBXN7 promotes macrophage infiltration and fibrosis of HK2 cells. **(A)** The expression of F4/80 in renal tissue of normal and DKD mice were measured by immunofluorescence (scale bar, 50 μm); **(B)** Morphology observation of macrophage cultured with HK2-cells under high and low glucose (scale bar, 100 μm); **(C)** The relative expressions of IL-6 and TNFα were detected in macrophage cells cultured with HK2-cells under high and low glucose by qRT-PCR; **(D)** The expressions of iNOS, CD86, IL-6, TNFα and CD206 in macrophages cultured with the supernatant of HK2 cells after overexpression of circUBXN7 and knockdown of circUBXN7 were determined by western blot; **(E)** Representative bright-field images of HK2 cells (scale bar, 50 μm) and HK2 cells stained with fluorescent phalloidin (scale bar, 20 μm) to show cell morphology after overexpression and knockdown of circUBXN7; **(F)** The expressions of EMT-related molecules in mRNA levels were detected by qRT-PCR after overexpression of circUBXN7 in HK2 cells under low glucose and knockdown of circUBXN7 in HK2 cells under high glucose; **(G, H)** The expressions of EMT-related proteins were determined after overexpression of circUBXN7 in HK2 cells cultured with low glucose and knockdown of circUBXN7 in HK2 cells cultured with high glucose by western blot **(G)** and by immunofluorescence **(H)**. scale bar, 100 μm; **(I)** The expression of EMT-related molecules in mRNA levels was detected by qRT-PCR after overexpression of circUBXN7 in HK2 cells under low glucose and knockdown of circUBXN7 in HK2 cells under high glucose; **(J, K)**. The expressions of fibrosis-related proteins were determined after overexpression of circUBXN7 in HK2 cells cultured with low glucose and knockdown of circUBXN7 in HK2 cells cultured with high glucose by western blot **(J)** and immunofluorescence **(K)**. scale bar, 100 μm). *P < 0.05, **P < 0.01, ***P < 0.001.

As the aforementioned result showed DKD-related circRNAs were enriched in immune inflammation and EMT-related signaling pathways, we further explore circUBXN7 role in macrophage activation, EMT and fibrosis of HK-2 cells using siRNAs targeting back-splicing site of circUBXN7 as well as overexpression lentivirus. The efficiencies of circUBXN7 over-expression and knockdown were assessed utilizing qRT-PCR. The findings revealed that circUBXN7 was significantly highly expressed when transfected with circUBXN7 lentivirus (**Figure S1A**) and down-expressed in cells transfected with circUBXN7 siRNAs (**Figure S1B**), while had no effect on the expression of linearUBXN7. Among them, the knockdown efficiency of si-circ3 was the best used for subsequent experiments. In addition, we found increased macrophage infiltration in the renal tubulointerstitial of db/db mice compared to the normal mice (**Figure 2A**). Macrophages exhibited pseudopodia and protrusions cultured with HK-2 cells under high glucose (**Figure 2B**). qRT-PCR results indicated the inflammatory cytokines expression of IL-6 and TNFα significantly increased in macrophages cultured with HK-2 cells under high glucose (**Figure 2C**). To investigate whether abnormal expression of circUBXN7 in HK2 cells affected macrophage polarization and secretion of inflammatory cytokines. We detected the M1 macrophage-related markers of iNOS, CD86, IL-6, TNFα and M2 macrophage-related marker of CD206 in macrophages co-cultured with HK2 cells after overexpression of circUBXN7 and knockdown of circUBXN7 by western blot. The expression of M1 macrophage markers was greatly increased but M2 macrophage marker was reduced in HK2-OE-circ group compared to HK2-OE-NC group, while the results were opposite in HK2-si-circ group compared to HK2-si-NC group (**Figure 2D**). These results demonstrated that upregulated circUBXN7 in HK-2 cells stimulated macrophages M1 polarization and suppressed macrophages M2 polarization.

Representative bright-field images and fluorescent phalloidin staining of HK-2 cells presented that HK-2 cells under low glucose had the typical characteristics of epithelial cells, such as cobblestone appearance, and HK-2 cells exhibited the typical characteristics of mesenchymal cells after overexpression of circUBXN7, such as spindle or elongated fusiform. Meanwhile, HK-2 cells appeared mesenchymal cell characteristics under high glucose, and the cells can return to an epithelial-like morphology after knocking down circUBXN7 (**Figure 2E**). Changes in cell morphology suggested that circUBXN7 can affect morphological alterations in HK-2 cells, and this change may be related to EMT. Next, E-cadherin (E-ca), Vimentin (Vim), and α-smooth muscle actin (α-SMA) as marker proteins of EMT were further detected in this study. qRT-PCR results displayed that the mRNA level of E-ca reduced, mRNA level of Vim and α-SMA raised after circUBXN7 overexpressed in HK-2 cells cultivated with low glucose. Conversely, the findings were reversed after the knockdown of circUBXN7 in HK-2 cells cultivated with high glucose (**Figure 2F**). Western blot (**Figure 2G**), and immunofluorescence (**Figure 2H**) results presented that E-ca protein expression reduced and protein expression of Vim and α-SMA increased after overexpression of circUBXN7 in HK-2 cells cultivated with low glucose. Meanwhile, the findings were reversed after the knockdown of circUBXN7 in HK-2 cells cultivated with high glucose.

Prior research has suggested that macrophage M1 polarization and the EMT process of renal tubular epithelial cells may produce fibrotic changes in DKD (8, 25, 26). To assess circUBXN7 effects on fibrosis, we examined collagenI (Col-I) and transforming growth factor β1 (TGFβ1), which have been reported as renal fibrosis markers in renal tubular epithelial cells. qRT-PCR exposed that mRNA expression of Col-I and TGFβ1were increased after overexpression of circUBXN7 in HK-2 cells under low glucose, as well as decreased after knockdown of circUBXN7 in HK-2 cells under high glucose (**Figure 2I**). Western blotting (**Figure 2J**) and immunofluorescence (**Figure 2K**) results indicated that Col-I and TGFβ1 increased after overexpression of circUBXN7 in HK-2 cells cultivated with low glucose, while the findings were reverses after knockdown of circUBXN7 in HK-2 cells cultivated with high glucose. These results recommend that ectopic expression of circUBXN7 in HK-2 cells promoted macrophage activation, inflammation, EMT and fibrosis *in vitro*.

### Transcript factor SP1 increases the production of circUBXN7 in renal tubular epithelial cells under high glucose condition

**Figure 3:**
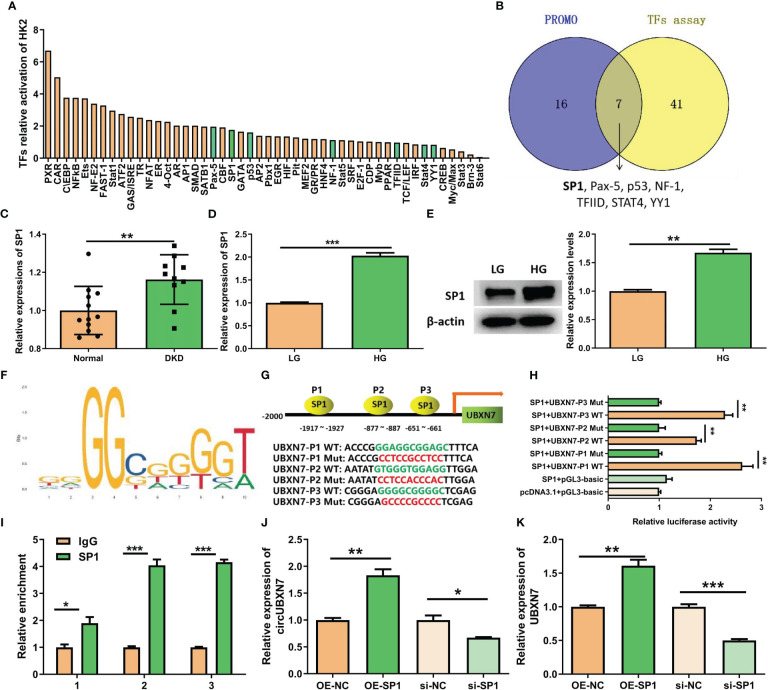
SP1 and UBXN7 promoter boost circUBXN7 expression. **(A)** The relative activation of multiple TFs was detected by TF activation profiling assay in HK2 cells under high glucose, and normalized by Myb; **(B)** Venn diagram constructed based on the TFs predicted by PROMO and the TFs assay. A total of 7 overlapping TFs (SP1, Pax-5, p53, NF-1, TFIID, STAT4, YY1) were found; **(C)** NephroSeq database showed the high expression of SP1 in the renal tubules of DKD patients; **(D)** SP1 was highly expressed in HK2 cells under high glucose detected by qRT-PCR; **(E)** Western blot displayed the SP1 protein level of HK2 cells cultured with low and high glucose; **(F)** JASPAR suggested that SP1 binds to the UBXN7 promoter; **(G)** Schematic model of wild type (WT) and mutant (Mut) sequences of three putative binding sites of SP1 on UBXN7 promoter; **(H)** Dual luciferase assay results presented that SP1 bind to WT UBXN7 promoter; **(I)** ChIP-qPCR analysis revealed that SP1 was enriched at the promoter region of UBXN7. **(J, K)** The expression levels of circUBXN7 **(J)** and UBXN7 **(K)** in HK2 cells were determined after SP1 up-regulation or down-regulation by qRT-PCR. *P < 0.05, **P < 0.01, ***P < 0.001.

Considering circUBXN7 up-regulation in DKD, it is interesting to explore how circUBXN7 was induced by high glucose. We performed a transcript factor activation profiling assay to detect the activation of transcript factors in HK-2 cells cultivated with high glucose, and 48 transcript factors were activated (**Figure 3A**). Meanwhile, 23 transcript factors were predicted to target the UBXN7 promoter through the PROMO database. Based on the results of transcript factors assay and PROMO, seven transcript factors (SP1, Pax-5, p53, NF-1, TFIID, STAT4, and YY1) were overlapped (**Figure 3B**). Among these DKD and UBXN7-related transcript factors, SP1 was focused on for further study according to our previous study in which SP1 promotes the mesangial cell proliferation in DKD (23). SP1 is of certain importance in establishing M1-like pro-inflammatory polarization (27). We noticed SP1 expression was elevated in the renal tissue of patients with DKD by Nephroseq database (https://www.nephroseq.org) (**Figure 3C**). Besides, qRT-PCR and western blot revealed that SP1 was markedly elevated in HK-2 cells cultivated with high glucose (**Figures 3D, E**). Then, JASPAR website supplied SP1 binding motif (**Figure 3F**) as well as expected three binding sites for SP1 in UBXN7 promoter; thus, these three binding sites of wild type (WT) or mutant (Mut) UBXN7 sequence were respectively incorporated into luciferase reporter plasmids (**Figure 3G**). The results of luciferase reporter demonstrated that SP1 boosted the luciferase activity of WT but not Mut in UBXN7 promoter (**Figure 3H**). Similarly, ChIP-qPCR assay demonstrated SP1 ability to bind to UBXN7 gene promoter and enhance its transcriptional activity (**Figure 3I**). To further study the impact of SP1 on circUBXN7 expression in HK-2 cells, we designed and synthesized SP1 overexpression plasmids and siRNAs. qRT-PCR and western blot was used to confirm transfection effectiveness (**Figures S1C–S1E**). qRT-PCR results revealed that up-regulated SP1 noticeably elevated expressions of UBXN7 and circUBXN7, while knockdown SP1 distinctly decreased levels of UBXN7 and circUBXN7 (**Figures 3J, K**). These findings imply that transcription factor SP1 could bind with UBXN7 promoter to drive the expression of circUBXN7.

### CircUBXN7 promotes fibrosis of HK-2 cells via IGF2BP2-dependent SP1 mRNA stability

**Figure 4:**
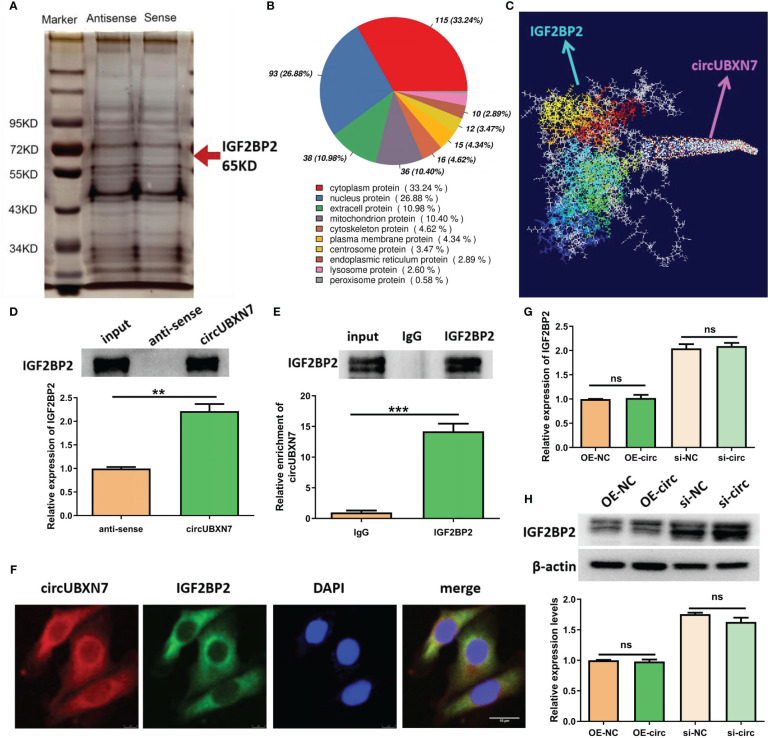
CircUBXN7 directly binds with IGF2BP2. **(A)** RNA pull-down experiment was performed using the specific circUBXN7 probe in HK2 cell lysates, followed by silver staining; **(B)** The subcellular localization of circUBXN7-specific binding proteins; **(C)** Alphafold and Rosetta software predicted the combination of circUBXN7 and IGF2BP2. **(D)** IGF2BP2 was detected after pull-down by western blot; **(E)** The enrichment of circUBXN7 with IGF2BP2 antibody compared with IgG, as determined by RIP assay; **(F)** FISH and IF assay indicated that circUBXN7 (red) was co-localized with IGF2BP2 (green) in the cytoplasm. Scale bar, 10 μm.; **(G)** The mRNA level of IGF2BP2 measured by qRT-PCR after overexpression and knockdown of circUBXN7; **(H)** The protein level of IGF2BP2 was detected by western blot after overexpression and knockdown of circUBXN7. **P < 0.01, ***P < 0.001, ns, no significance.

Combined with the results of GO analysis and the sub-location of circUBXN7, our data revealed DKD-related circRNAs were mainly involved in protein binding and circUBXN7 dominantly located in HK-2 cells cytosol, suggesting circUBXN7 may play a role in DKD by targeting for protein in HK-2 cells cytosol. To investigate the molecular mechanism of circUBXN7, we adopted RNA pull down, then silver staining (**Figure 4A**) and mass spectrometry to determine circUBXN7-binding proteins; we found that most proteins localized in the cytoplasm accounted for 33.24% (**Figure 4B**). Among these proteins, we showed top 10 proteins bound by circUBXN7 (**Table 2**) and IGF2BP2 was the top one of mass spectrometry scores. Moreover, Alphafold and Rosetta software displayed the predicted combination of circUBXN7 and IGF2BP2 (**Figure 4C**). Western blot results validated IGF2BP2 was also found to be significantly enriched on circUBXN7 (**Figure 4D**). RIP assay demonstrated that IGF2BP2 could specifically bind with circUBXN7 in HK-2 cells (**Figure 4E**). FISH and IF co-staining assay exhibited that circUBXN7 and IGF2BP2 were co-localized in cytosol (**Figure 4F**). Although these findings imply that circUBXN7 can directly bind to IGF2BP2, qRT-PCR (**Figure 4G**) and western blot (**Figure 4H**) results exhibited that upregulation and downregulation of circUBXN7 had not altered the transcript and protein level of IGF2BP2, suggesting that circUBXN7 was irresponsible for the expression level of IGF2BP2 via their binding.

**Table 2:** Top 10 proteins bound by circUBXN7 detected by mass spectrometry.

Gene	Description	MW [kDa]	Coverage [%]	Unique Peptides	Score
IGF2BP2	Insulin-like growth factor 2 mRNA-binding protein 2	66.7	15	7	15.51
LARP1	La-related protein 1	123.4	5	6	9.99
RPS3	40S ribosomal protein S3	26.7	22	5	9.23
PABPC4	Polyadenylate-binding protein	72.3	12	5	15.13
IGF2BP3	Insulin-like growth factor 2 mRNA-binding protein 3	63.7	13	5	12.13
RPS3A	40S ribosomal protein S3a	29.9	18	5	13.37
MTDH	Metadherin, isoform CRA a	63.8	10	5	12.53
IGF2BP1	Insulin-like growth factor 2 mRNA-binding protein 1	63.4	9	5	9.49
FAM120A	Constitutive coactivator of PPAR-gamma-like protein 1	121.8	6	5	14
FLG	Truncated profilaggrin	294.3	3	5	15.09

**Figure 5:**
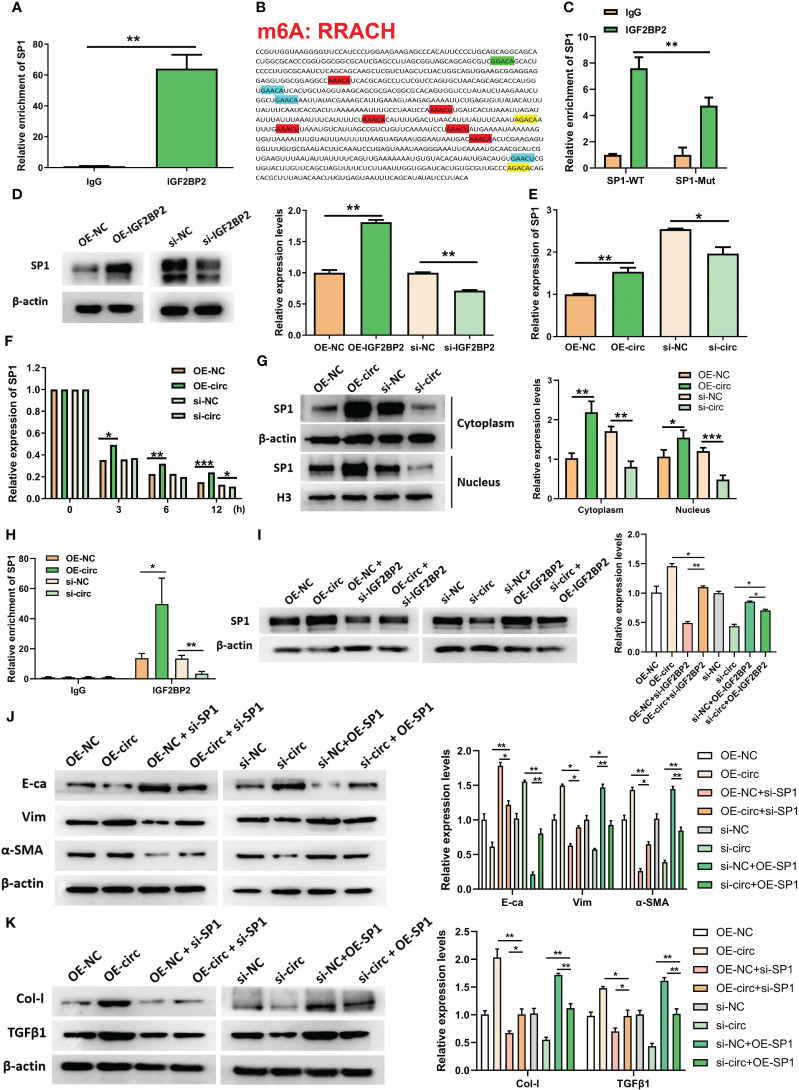
CircUBXN7 stabilizes SP1 mRNA expression by recruiting IGF2BP2 and promotes fibrosis in HK2 cells. **(A)** The mRNA of SP1 was significantly enriched in the anti-IGF2BP2 by RIP; **(B)** m6A motif site on SP1 mRNA (RRACH: R = G, A; H = A, C, U); **(C)** The change of SP1 enrichment in anti-IGF2BP2 after mutating the m6A motif of SP1 by RIP; **(D)** The protein level of SP1 assessed in HK2 cells following overexpression and knockdown of IGF2BP2 by western blot; **(E)** The mRNA level of SP1 was measured by qRT-PCR following overexpression and knockdown of circUBXN7; **(F)** Actinomycin D treatment followed by qRT-PCR analyzed the stability of SP1 after overexpression and knockdown of circUBXN7; **(G)** Western blot detected SP1 expression in cytoplasm and nucleus after overexpression and knockdown of circUBXN7; **(H)** The change of SP1 enrichment in anti-IGF2BP2 after overexpression and knockdown of circUBXN7 by RIP. **(I)** CircUBXN7 recruited IGF2BP2 to regulate SP1 expression; **(J)** The rescue experiment was performed to detect the expression level of E-ca, Vim, and α-SMA in HK2 cells cultured with low glucose after overexpression of circUBXN7 or knockdown of SP1, and in HK2 cells cultured with high glucose after knockdown of circUBXN7 or overexpression of SP1; **(K)** The expression level of Col-I and TGFβ1 in HK2 cells cultured with low glucose after overexpression of circUBXN7 or knockdown of SP1 and in HK2 cells cultured with high glucose after knockdown of circUBXN7 or overexpression of SP1. *P < 0.05, **P < 0.01, ***P < 0.001.

Since IGF2BP2 serves as an established m6A reader in diseases, we explored whether circUBXN7 affects the m6A reading ability of IGF2BP2. To further investigate the interaction between circUBXN7 and IGF2BP2 in DKD, we explored whether circUBXN7 affects the m6A reading ability of IGF2BP2. First, we screened the IGF2BP2 m^6^A targets by m6A2Target. Surprisingly, SP1 was one of the potential targets. Combined with RIP experiments, we confirmed that the mRNA of SP1 was significantly enriched in anti-IGF2BP2 (**Figure 5A**). Subsequently, we discovered that the SP1 had six m6A consensus sequences (–GGACA–, –GAACA–, –AAACT–, –AGACA–, –GAACA–, –GAACT–; WT) (**Figure 5B**). And we mutated those m6A sites to obtain a SP1 mutant (Mut). RIP assay suggested IGF2BP2 could bind significantly more with SP1 WT than SP1 mutant (**Figure 5C**). To verify whether IGF2BP2 could regulate SP1, we created and constructed IGF2BP2 overexpression plasmids and siRNAs, as well as qRT-PCR and western blot was utilized to determine transfection efficiency (**Figure S1F–S1H**). SP1 was obviously elevated after overexpression of IGF2BP2 and declined after the intervention of IGF2BP2 (**Figure 5D**). Indeed, we found that enforced expression of circUBXN7 promoted the expression and stability of SP1 mRNA. Meanwhile, loss of circUBXN7 inhibited the expression and stability of SP1 mRNA via qRT-PCR (**Figures 5E, F**). CircUBXN7 overexpression improved the protein level of SP1 in the cytoplasm and activated SP1 expression in the nucleus, while depletion of circUBXN7 suppressed the effect (**Figure 5G**). RIP experiments revealed that the enrichment of SP1 mRNA in the IGF2BP2 was significantly increased after circUBXN7 overexpression, while it was substantially weakened by the knockdown of circUBXN7 (**Figure 5H**). Additionally, western blot results suggested that enforced expression of circUBXN7 promoted the expression of SP1 could be rescued by silencing of IGF2BP2. Meanwhile, circUBXN7 knockdown effect on inhibition of SP1 expression could be rescued by IGF2BP2 over-expression (**Figure 5I**). The above results displayed that circUBXN7/IGF2BP2/SP1 formed RNA-protein complex, which improves mRNA stability of SP1 and the expression of SP1.

Additionally, results presented circUBXN7 overexpression improved EMT expression and fibrosis-related proteins could be rescued after knockdown of SP1, while the effect of silencing of circUBXN7 undermined expression of EMT and fibrosis-related proteins could be neutralized by over-expression of SP1 (**Figures 5J, K**). Considering SP1 induced the transcriptional activation of circUBXN7 in this study, we concluded that circUBXN7 and SP1 formed a positive feedback loop, regulating EMT and fibrosis of renal tubular epithelial cells under high glucose condition.

### CircUBXN7 aggravates DKD progression *in vivo*

**Figure 6:**
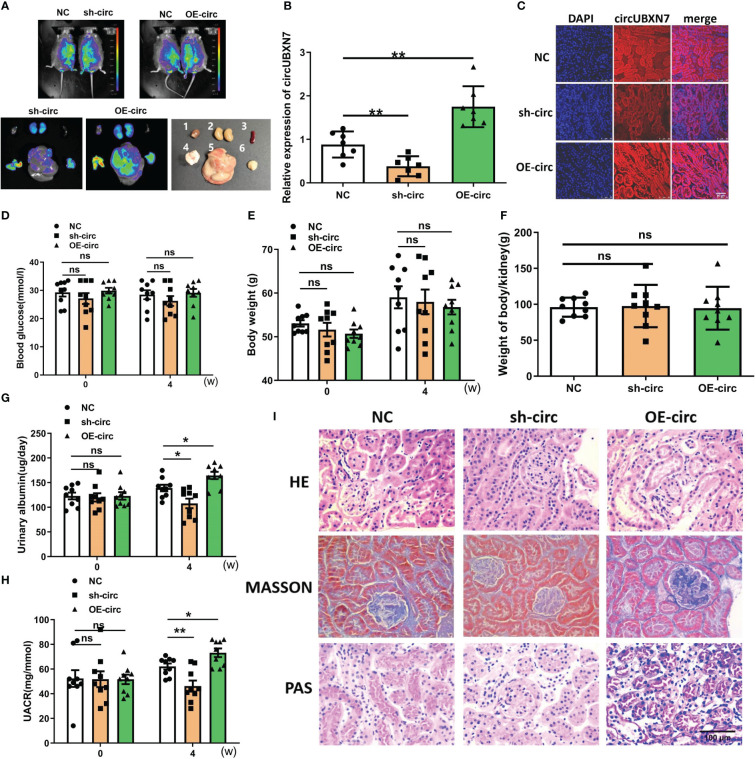
Overexpression of circUBXN7 aggravates DKD progression *in vivo*. **(A)** Representative bioluminescent imaging displayed the efficiency of lentivirus infection in db/db mice (1: heart; 2: kidney; 3: spleen; 4: pancreas; 5: liver; 6: muscle); **(B)** The expression of circUBXN7 in DKD kidney tissue infected lentivirus detected by qRT-PCR; **(C)** FISH showed the expression of circUBXN7 in DKD kidney tissue. Scale bar, 50 μm; **(D–F)** The changes of blood glucose **(D)**, body weight **(E)**, and weight of body/kidney **(F)**; after lentivirus infection of overexpression and knockdown of circUBXN7; **(G)** Urinary albumin changes after four weeks of lentivirus infection; **(H)** UACR changes after four weeks of lentivirus infection; **(I)** HE, PAS, and Masson staining showed the changes in kidney tissue upon circUBXN7 overexpression or knockdown. Scale bar, 100 μm. n=7~9 per group. *P < 0.05, **P < 0.01, ns, no significance.

To further confirm the role of cirUBXN7 in DKD, lentivirus was injected into the tail vein of db/db mice to raise or reduce circUBXN7 expression in kidneys. Besides, we found that the conservation of circUBXN7 was as high as 91% between humans and mice. Representative bioluminescent imaging of green fluorescence in kidneys displayed lentivirus can be brought successfully into the kidneys (**Figure 6A**). The results of renal tissue qRT-PCR (**Figure 6B**) and FISH (**Figure 6C**) indicated circUBXN7 overexpression and knockdown lentivirus were able to efficiently upregulate and downregulate circUBXN7 expression in renal tissue of db/db mice, respectively.

To assess circUBXN7 role *in vivo*, we detected the effects of circUBXN7 on renal function, blood glucose, body weight, and the weight of body/kidney of db/db mice treated with circUBXN7 overexpression and knockdown lentivirus. Although blood glucose, body weight, and the weight of body/kidney displayed no significant difference in the groups (**Figures 6D, F**), albuminuria was found to be regulated by circUBXN7. Results indicated that downregulated circUBXN7 reduced 24 h urinary albumin and UACR, while upregulated circUBXN7 elevated 24 h urinary albumin and UACR (**Figures 6G, H**). Moreover, HE, Masson, and PAS staining revealed that circUBXN7 significantly enhanced tubular hypertrophy, tubular basement membrane thickening, and renal interstitial fibrosis (**Figure 6I**). Therefore, the restoration of circUBXN7 expression attenuated the progression of DKD and upregulated circUBXN7 aggravated the development of DKD.

### CircUBXN7 promotes macrophage infiltration and fibrosis of renal tissue *in vivo*

**Figure 7:**
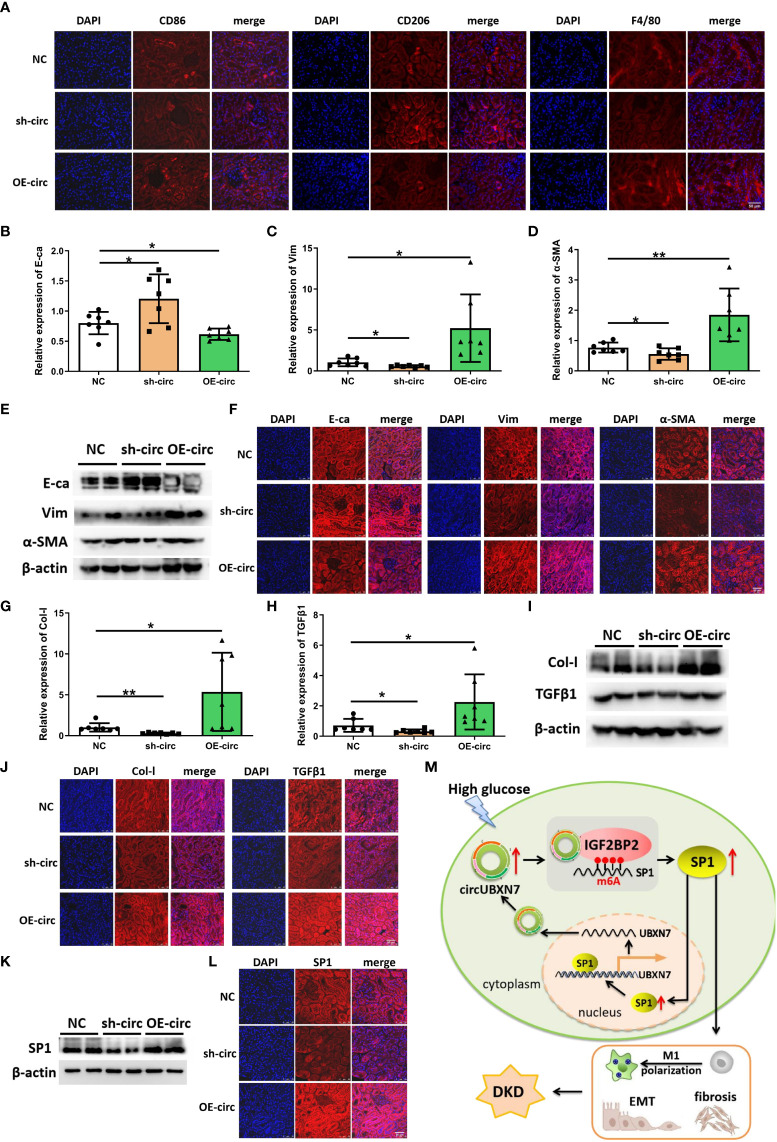
Overexpression of circUBXN7 promotes macrophage infiltration and fibrosis *in vivo*. **(A)** The expression of CD86, CD206 and F4/80 was detected by immunofluorescence in DKD mice upon circUBXN7 overexpression or knockdown, respectively. Scale bar, 50 μm; **(B–D)** The expression of E-ca **(A)**, Vim **(B)**, and α-SMA **(C)** in mRNA levels were assessed by qRT-PCR following circUBXN7 overexpression and knockdown among each group of db/db mice; **(E, F)** The expression of EMT-related proteins was detected by western blot **(E)** and immunofluorescence **(F)** upon circUBXN7 overexpression or knockdown, respectively. Scale bar, 50 μm. **(G, H)** The expression of Col-I **(G)** and TGFβ1 **(H)** in mRNA levels were detected by qRT-PCR after overexpression and knockdown of circUBXN7 in each group of db/db mice; **(I, J)**. Col-I and TGFβ1 expression was detected by western blot **(I)** and immunofluorescence **(J)** upon circUBXN7 overexpression or knockdown, respectively. Scale bar, 50 μm; **(K, L)** Western blot **(K)** and immunofluorescence **(L)** showed the expression of SP1 in DKD mice after overexpression and knockdown of circUBXN7. Scale bar, 50 μm; **(M)** Schematic diagram depicting circUBXN7 generation and mechanisms of how circUBXN7 aggravates DKD progression. *P < 0.05, **P < 0.01.

To understand whether circUBXN7 affects macrophage infiltration, renal EMT and fibrosis of db/db mice, we explore circUBXN7 role in changes of macrophage marker (F4/80), M1 macrophage marker (CD86), M2 macrophage marker (CD206), E-ca, Vim, α-SMA, Col-I and TGFβ1 in the kidney. We found that there was a small amount of macrophage infiltration in the renal tubulointerstitial of control db/db mice, and the amount of macrophage infiltration was decreased with the downregulation circUBXN7, while upregulated circUBXN7 significantly aggravated macrophage infiltration, especially M1 macrophages, and inhibited M2 macrophage infiltration (**Figure 7A**). Then, qRT-PCR results suggested that the expression of epithelial marker E-ca was markedly elevated, and mesenchymal marker Vim and α-SMA were markedly decreased in db/db mice with the downregulation circUBXN7. Meanwhile, upregulated circUBXN7 significantly reversed these changes (**Figures 7B, D**). Western blot and immunofluorescence results were consistent with the qRT-PCR consequence (**Figures 7E, F**). Likewise, qRT-PCR showed that Col-I and TGFβ1 expression in the kidney was significantly reduced after the knockdown of circUBXN7. Meanwhile, the expression of Col-I and TGFβ1 was significantly elevated after overexpression of circUBXN7 (**Figures 7G, H**). Western blot and immunofluorescence observation were consistent with the qRT-PCR results (**Figures 7I, J**). Additionally, western blot and immunofluorescence presented that deficiency of circUBXN7 attenuated the expression of SP1, and the augmented circUBXN7 enhanced the expression of SP1 *in vivo* (**Figures 7K–L**). This novel DKD-related circRNA circUBXN7 acts as an essential driver for EMT and fibrosis in DKD *in vitro* and *in vivo* through the positive loop with SP1 via direct targeting for the m6A reader IGF2BP2 (**Figure 7M**). These results display that circUBXN7 has a significant function in macrophage-induced renal EMT and tubulointerstitial fibrosis of diabetic mice.

## Discussion

CircRNAs have been identified as crucial regulators of pathological processes and diseases, including DKD. In this study, we found circUBXN7 as a significant signature circRNA in the plasma of DKD patients, which was correlated with renal function in DKD patients. The expression of circUBXN7 was transcriptionally modulated by SP1 in HK-2 cells cultivated under high glucose conditions. Furthermore, circUBXN7 overexpression in HK-2 cells was linked with macrophage activation, EMT and fibrosis of HK-2 cells *in vitro*. Furthermore, DKD model mice infected with circUBXN7 overexpression and knockdown lentivirus confirmed the effect of circUBXN7 on renal function and renal fibrosis *in vivo*. Our results exposed the function of circUBXN7 induced by high glucose in the progression of DKD, highlighting circUBXN7 may tremendously be a potential candidate for diagnostic biomarkers and therapeutic targets of DKD.

CircRNAs, as stable non-coding RNA, have been widely reported as plasma biomarkers in some complex diseases (28, 29). Most circRNAs are found in cytosol, which increases the likelihood of their early release in response to tissue injury (30). Therefore, circRNAs microarray was performed to detect circRNAs expression profile in the plasma of DKD patients. We found circUBXN7 was significantly upregulated in DKD plasma. Besides, circUBXN7 expression in plasma was positively linked with UACR and 24 h urinary albumin of DKD patients. It is widely known that the changes in UACR are sensitive indices for the diagnosis of diabetic renal injury (31). This evidence proves that circUBXN7 may be a new plasma biomarker for the primary detection of DKD. Certainly, as a plasma biomarker, we still need to collect more samples to verify it.

Additionally, EMT in tubular epithelial cells is essential for the progress of DKD-related renal interstitial fibrosis (32). Besides, renal interstitial fibrosis is characterized by fibrosis-related protein deposition in the renal cortex (33). In our study, The EMT program and fibrosis process of HK-2 cells were observed to be regulated by abnormal expression circUBXN7 *in vivo* and *in vitro*. Therefore, these results show that circUBXN7 plays a direct protective function in EMT process and renal interstitial fibrosis in DKD.

Macrophages play a key role in driving inflammation and promoting renal fibrosis by producing inflammatory cytokines and acting in a paracrine manner (34). Consistently, during the course of circUBXN7-induced renal fibrosis, we observed increasing inflammatory cytokines level of IL-6 and TNFα in macrophages cultured with HK-2 cells of circUBXN7 overexpression, suggesting macrophage activation might accelerate EMT and fibrosis HK-2 cells via inflammatory cytokines secretion. Inflammation is important for HK-2 cells-induced macrophages infiltration and for macrophage-mediated EMT and fibrosis of HK-2 cells, circUBXN7 may become a critical regulator for the crosstalk between HK-2 cells and macrophages. Despite several studies demonstrating that abnormally expressed molecules in tubular epithelial cells provokes macrophage infiltration to aggravate interstitial fibrosis, how this signals communications between the tubular epithelial cells and macrophages has remained obscure. It will be the main focus of our subsequent research.

It is worth noting that circRNAs may also serve as scaffolds aiding the construction of protein complexes (35), transcriptional regulators (36), RBP-binding partners (37), and translational templates (38). It was reported that numerous circRNAs could regulate the development of DKD by acting as competing endogenous RNAs (ceRNAs) (39, 40). Furthermore, proteins are direct effectors of almost all essential activities in the process of diseases (41). These reports suggested that circRNAs might have crucial functions in the pathogenesis of DKD. However, there are few studies on regulating protein function by circRNA-binding protein in the process of DKD. IGF2BP2 is involved in regulating the location and stability of mRNA. As RBP, IGF2BP2 binds with many RNAs and recruits these transcripts into cytoplasmic protein-RNA complexes as RBP (42). IGF2BP2 can specifically act as an m6A reader, a prevalent RNA modification that has been demonstrated to have basic effects on numerous cellular processes (43). IGF2BP2 stabilizes target mRNA by acting as an m6A reader in various biological processes and disease development, as supported by accumulating data. For example, IGF2BP2 maintains FEN1 expression through an m6A-dependent mechanism to promote liver cancer growth (44). m6A Reader IGF2BP2 controls macrophage phenotypic activation and inflammatory diseases by enhancing the stability of TSC1 and PPARγ (45). LINC00460/DHX9/IGF2BP2 complex stimulates colorectal cancer proliferation and metastasis by interacting with IGF2BP2 and regulating HMGA1 mRNA stability by m6A modification (46). Nevertheless, the biological function and underlying mechanisms of IGF2BP2 in DKD are still largely elusive.

Herein, we discovered a distinct circRNAs mechanism in DKD, that circUBXN7 triggered by high glucose could specially bind with m6A reader IGF2BP2 to be involved in posttranscriptional modulation of SP1 gene by stabilizing SP1 mRNA. By utilizing m6A2Target and RIP assay, we found that SP1 mRNA was significantly enriched to m6A reader IGF2BP2 in HK-2 cells. Further, we confirmed IGF2BP2 recognized and bound to SP1 mRNA in an m6A-dependent manner. As anticipated, we found that upregulated circUBXN7 enhanced the binding of IGF2BP2 and SP1 mRNA by RIP. In this process, we discovered circUBXN7/IGF2BP2/SP1 formed RNA-protein complex, which improved the mRNA stability and expression of SP1. Interestingly, SP1 expression was obviously increased in the renal tissue of patients with DKD and HK-2 cells under high glucose conditions. SP1 is one of these ubiquitous and multifunctional TFs from Sp/Kruppel-like family (KLF) TFs that are the predominant types of zinc-finger DNA-binding proteins (47). Meanwhile, we noticed that SP1 induced the transcription of circUBXN7 as a transcription factor by binding to the GGAGGCGGAGC, GTGGGTGGAGG, and GGGGCGGGGC sequences in the promoter region of UBXN7 gene. Moreover, it has been reported that SP1 contributes to EMT (48, 49) and extracellular matrix production (50) in tubular epithelial cells in DKD, worsening the tubular injury. Silencing of SP1 enhanced the M1 polarization of macrophages in murine lung tissues of Type 2 diabetes mellitus (51). Nevertheless, the biological function and probable mechanisms of SP1 in DKD require additional investigation. Generally, circUBXN7 and SP1 form a positive feedback regulatory pathway to influence the occurrence and development of DKD. It provides a novel regulatory network of circRNA in the post-transcriptional regulation of DKD events. To sum up, we discovered circUBXN7/SP1 formed a positive feedback loop mediated by IGF2BP2, subsequently promoting tubular EMT and fibrosis in DKD. The rescue experiments further validated that circUBXN7 could regulate SP1 expression by the interaction with IGF2BP2. Our result provides new evidence for abnormal expression of SP1 at post-transcriptional regulatory level by circUBXN7-IGF2BP2 complexes.

## Conclusion

In summary, our study provides the first evidence that circUBXN7/IGF2BP2/SP1 formed RNA-protein complex, which improves mRNA stability of SP1 and the expression of SP1, promoting macrophage infiltration and renal fibrosis, accelerating the progression of DKD. The circUBXN7 may represent an effective therapeutic approach for DKD.

## Data availability statement

The datasets presented in this study can be found in online repositories. The names of the repository/repositories and accession number(s) can be found below: GSE231923 (GEO).

## Ethics statement

The studies involving human participants were reviewed and approved by the Second Affiliated Hospital of Chongqing Medical College (2018146). The patients/participants provided their written informed consent to participate in this study. The animal study was reviewed and approved by the Ethical Committee of Chongqing Medical University (2022135). Written informed consent was obtained from the individual(s) for the publication of any potentially identifiable images or data included in this article.

## Author contributions

ZZ and YS designed the research and supervised the project. ZL and DL performed all experiments and constructed the manuscript. XL was responsible for the collecting of clinical samples and provided support for animal investigations. ZL analyzed the data statistically. All bioinformatics work was done by RP and HL. Technical assistance for experiments was supplied by TW and KW. ZL, ZZ, and YS supervised all studies and wrote the manuscript. All authors contributed to the article and approved the submitted version.
