# Supplementary material for: CircUBXN7 promotes macrophage infiltration and renal fibrosis associated with the IGF2BP2-dependent SP1 mRNA stability in diabetic kidney disease
Source: Front Immunol. 2023 Sep 6;14:1226962. doi: 10.3389/fimmu.2023.1226962 (PMC10516575; doi:10.3389/fimmu.2023.1226962)
Supplement: Supplementary file 1 [file DataSheet_1.pdf]

Supplementary Material

CircUBXN7 promotes macrophage infiltration and renal fibrosis associated with the IGF2BP2-dependent SP1 mRNA stability in DKD

Ziyue Lin, Dan Lv, Xiaohui Liao, Rui Peng, Handeng Liu, Tianhui Wu, Keqian Wu, Yan Sun*, Zheng Zhang*

*** Correspondence:** Corresponding Author: Yan Sun, yansun@cqmu.edu.cn and Zheng Zhang, zhangzheng@cqmu.edu.cn

1 Supplementary Figures

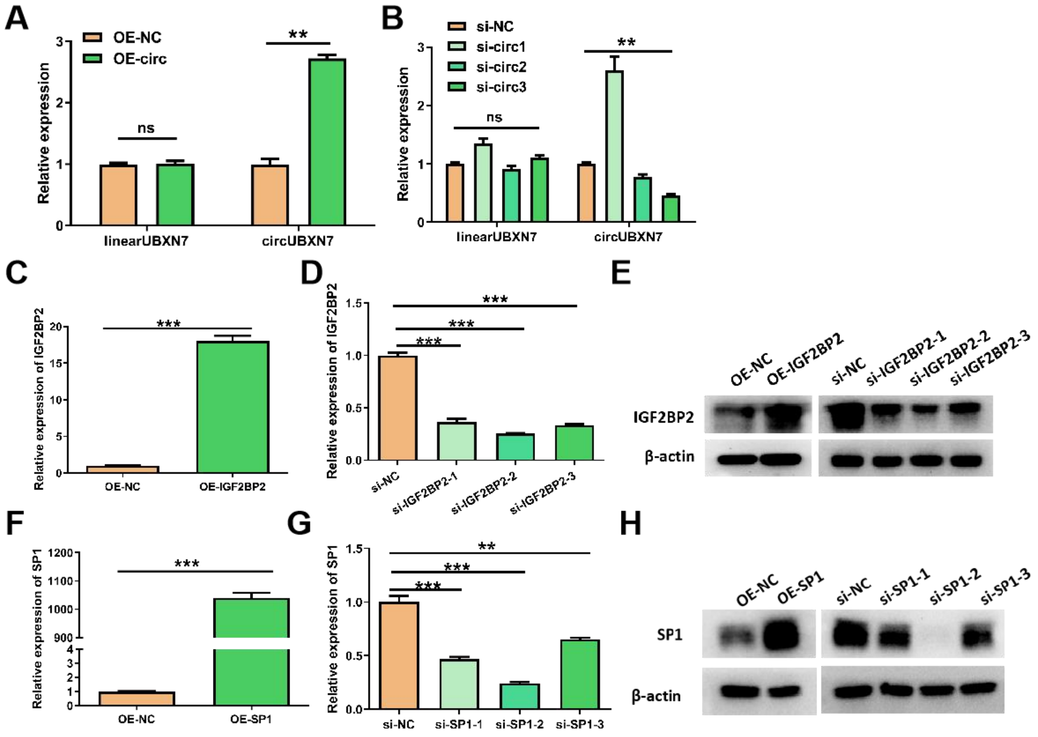

Figure S1. Validation of over-expression lentivirus/plasmid and siRNAs. A. The relative expressions of circUBXN7 and linearUBXN7 were detected in HK2 cells after transfection of circUBXN7 overexpressed lentivirus by qRT-PCR; B. qRT-PCR analysis of circUBXN7 and linearUBXN7 expression in HK2 cells transfected with siRNAs; C-D. The relative expression of SP1 were detected in HK2 cell after transfection of SP1 overexpressed plasmid (C) and siRNAs (D) by qRT-PCR. E. The relative expression of SP1 were detected after transfection of SP1 overexpressed plasmid and

siRNAs by western blot. F-G. The relative expression of IGF2BP2 was detected after transfection of IGF2BP2 overexpressed plasmid (F) and siRNAs (G) by qRT-PCR. H. The relative expression of IGF2BP2 was detected after transfection of IGF2BP2 overexpressed plasmid and siRNAs by western blot. Data are shown as mean ± SD and representative of three independent experiments. **P < 0.01, ***P < 0.001.

2 Supplementary Tables

Table S1. Primer sequence

Gene (human)	Sequence
circJA760602（1）	F: TGTAGACCTACTTGCGCTGC<br>R: CAACTCCTACATACTTCCCCCA
circSNX13	F: TCCCCCTGGACAGCATTCTT<br>R: TCTGACTGCTTCTAGCTCTCCA
circMTND5	F: AGCAGGAATACCTTTCCTCACAG<br>R: GCCAATGGTGAGGGAGGTTG
circJA760600	F: TGTAGACCTACTTGCGCTGC<br>R: TCATTGGACAAGTAGCATCCGT
circJA760602（2）	F: TGGTGGGCCATACGGTAGTATT<br>R: TCTTCCCACTCATCCTAACCC
circDCAF6	F: ACAGTGATGATGACCCAGTCC<br>R: GTTCCTTCTGTGCTGTAGTGC
circHSPA12A	F: TCGACTTTGGGACCACATCC<br>R: GGGAGCCGTTTCTCATCACAT
circUBXN7	F: GCAGCTTTGAAACAAGAAGAAGT<br>R: TGCAGGCCGTCGTCTTTTAG
circCOQ4	F: TCACAGGAGTGAGGGCTACA<br>R: CTGCGGGGCAAGAAAAATTGA
UBXN7	F: GGACTTTCTAGCAGTCCCCC<br>R: GTAAGGAGGCTCTGATGGCA
β-actin	F: GAGAAAATCTGGCACCACACC<br>R: GGATAGCACAGCCTGGATAGCAA
E-ca	F: GCTTCAGCAAAGACAACGAG<br>R: GTGTAATGCAGGACCACAGC
Vim	F: CGTGATGCTGAGAAGTTTCGT<br>R: TGGATTCACTCCCTCTGGTTG
α-SMA	F: TACTACTGCTGAGCGTGAGA<br>R: CATCAGGCAACTCGTAACTC
Col-I	F: GAGGGCCAAGACGAAGACATC<br>R: CAGATCACGTCATCGCACAAC
TGFβ1	F: GCTAAGGCGAAAGCCCTCAAT<br>R: CCTGGCGATACCTCAGCAACC
UBXN7-P1	F: TCAACATGGTGAAACCTCGTC

	R: TTACTGAAAGCTCCGCCTC
UBXN7-P2	F: TCACGCCTGTAATCCCAACA<br>R: CCTTCCAACCTCCACCCAC
UBXN7-P3	F: GCAACCGAGCAGCCAACCC<br>R: ATTGACCGCCCCTCGAGCC
SP1	F: TGGCAGCAGTACCAATGGC<br>R: CCAGGTAGTCCTGTCAGAACTT
IGF2BP2	F: AGCTAAGCGGGCATCAGTTTG<br>R: CCGCAGCGGGAAATCAATCT
**Gene (mouse)**	**Sequence**
E-ca	F: CCACCAGATGACGATACCCG<br>R: GCTTCAGAACCACTCCCCTC
Vim	F: TGAGATCGCCACCTACAGGA<br>R: GAGTGGGTGTCAACCAGAGG
α-SMA	F: GAGGCACCACTGAACCCTAA<br>R: CATCTCCAGAGTCCAGCACA
Col-I	F: ATCTCCTGGTGCTGATGGAC<br>R: ACCTTGTTTGCCAGGTTCAC
TGFβ1	F: ATGGTGGACCGCAACAAC<br>R: CCAAGGTAACGCCAGGAA
SP1	F: AAGGATGCGGCAAAGTAT<br>R: CGTCCGAACGTGTAAAGC
TNFα	F: CAGCCTCTTCTCATTCCTGC<br>R: GGTCTGGGCCATAGAACTGA
IL-6	F: GATGGATGCTACCAAACTGGA<br>R: TCTGAAGGACTCTGGCTTTG
β-actin	F: TCCATCATGAAGTGTGACGT<br>R: GAGCAATGATCTTGATCTTCAT

Table S2. siRNA and probe sequences

Probe name	Sequence
circUBXN7	5'Cy3-GAACTTCTTCTTGTTTCAAAGCTGCCTTT-3'Cy3
Antisense	5'Cy3-AAAGGCAGCTTTGAAACAAGAAGAAGTTC-3'Cy3
**siRNA name**	**Sequence**
si-circ1	GGCAGCTTTGAAACAAGAA
si-circ2	AGCTTTGAAACAAGAAGAA
si-circ3	GAAACAAGAAGAAGTTCGT
si-SP1-1	GCAACATCATTGCTGCTAT
si-SP1-2	GCCAATAGCTACTCAACTA
si-SP1-3	CTCCCAACTTACAGAACCA
si-IGF2BP2-1	CATGCCGCATGATTCTTGA

si-IGF2BP2-2	GAACGAACTGCAGAACTTA
si-IGF2BP2-3	AACAGGGACCAAGATAACA
